# Early responses of insulin signaling to high-carbohydrate and high-fat overfeeding

**DOI:** 10.1186/1743-7075-6-37

**Published:** 2009-09-28

**Authors:** Rebecca L Adochio, J Wayne Leitner, Karen Gray, Boris Draznin, Marc-Andre Cornier

**Affiliations:** 1 Department of Medicine, University of Colorado Denver, Aurora, Colorado, USA; 2 Research Service, Department of Veterans Affairs, Denver, Colorado, USA

## Abstract

**Background:**

Early molecular changes of nutritionally-induced insulin resistance are still enigmatic. It is also unclear if acute overnutrition alone can alter insulin signaling in humans or if the macronutrient composition of the diet can modulate such effects.

**Methods:**

To investigate the molecular correlates of metabolic adaptation to either high-carbohydrate (HC) or high-fat (HF) overfeeding, we conducted overfeeding studies in 21 healthy lean (BMI < 25) individuals (10 women, 11 men), age 20-45, with normal glucose metabolism and no family history of diabetes. Subjects were studied first following a 5-day eucaloric (EC) diet (30% fat, 50% CHO, 20% protein) and then in a counter balanced manner after 5 days of 40% overfeeding of both a HC (20% fat, 60% CHO) diet and a HF (50% fat, 30% CHO) diet. At the end of each diet phase, *in vivo *insulin sensitivity was assessed using the hyperinsulinemic-euglycemic clamp technique. *Ex vivo *insulin action was measured from skeletal muscle tissue samples obtained 15 minutes after insulin infusion was initiated.

**Results:**

Overall there was no change in whole-body insulin sensitivity as measured by glucose disposal rate (GDR, EC: 12.1 ± 4.7; HC: 10.9 ± 2.7; HF: 10.8 ± 3.4). Assessment of skeletal muscle insulin signaling demonstrated increased tyrosine phosphorylation of IRS-1 (p < 0.001) and increased IRS-1-associated phosphatidylinositol 3 (PI 3)-kinase activity (p < 0.001) following HC overfeeding. In contrast, HF overfeeding increased skeletal muscle serine phosophorylation of IRS-1 (p < 0.001) and increased total expression of p85α (P < 0.001).

**Conclusion:**

We conclude that acute bouts of overnutrition lead to changes at the cellular level before whole-body insulin sensitivity is altered. On a signaling level, HC overfeeding resulted in changes compatible with increased insulin sensitivity. In contrast, molecular changes in HF overfeeding were compatible with a reduced insulin sensitivity.

## Background

Overnutrition associated with weight gain can lead to obesity and insulin resistance [1-6]. Eventually individuals can develop type 2 diabetes mellitus (T2DM) and cardiovascular disease leading to a significant increase in morbidity and mortality. We aimed to unravel the earliest molecular changes associated with the development of insulin resistance as a result of overnutrition and to determine if acute bouts of caloric excess, before weight gain occurs, can lead to changes in insulin signaling.

There is a paucity of literature studying short-term overfeeding of normal lean individuals. Animal studies have shown that overfeeding can induce insulin resistance acutely [7]. Human studies have shown that varying amounts and duration of overfeeding can lead to elevations in fasting insulin levels in the setting of normoglycemia [8-14]. Our group has previously found that 3 days of overfeeding (50% caloric excess) in lean healthy individuals led to a significant decrease in whole-body insulin sensitivity [15,16]. Most recently, Brøns, et al. found that 5 days of high-fat overfeeding (50% caloric excess) in lean individuals resulted in no change in whole-body insulin sensitivity as measured by M-value and Glucose Disposal Rate (GDR) [17]. Therefore, it remains unclear if acute overnutrition alone can induce insulin resistance in humans. If acute overfeeding can impact insulin sensitivity, the next question is whether the effect is altered by the macronutrient content of the consumed diet. Although many studies in the literature have assessed macronutrient effects on insulin sensitivity [18-22] these studies were not performed in the setting of overfeeding. Population studies have shown that diets rich in fat appear to be associated with development of insulin resistance, obesity and T2DM [23,24]. There have also been reports that diets rich in carbohydrates with a high glycemic index may be associated with increased hepatic glucose production and the development of T2DM [25,26].

The potential link between energy intake and changes in insulin action remain unclear. At a whole body level, insulin resistance can be defined when higher than normal concentrations of insulin are necessary to maintain euglycemia. On a cellular level, metabolic insulin resistance is known to display a reduced strength of signaling via the insulin receptor substrate (IRS)-phophatidylinositol (PI) 3-kinase pathway. In almost all cases of insulin resistance there is a decline in PI 3-kinase activity [27-29]. Two complementary mechanisms have emerged as potential explanations for the reduced strength of the IRS-PI 3-kinase signaling pathway. First, PI 3-kinase activity is minimized secondary to serine phosphorylation of IRS proteins by intracellular signaling intermediates such as mTOR-p70 S6 (S6K1) kinase-dependent mechanism or other kinases (JNK, IKKβ, or PKC). Serine phosphorylation of IRS proteins results in a diminished ability of IRS proteins to attract PI 3-kinase [30-37]. In response to insulin and amino acids mammalian target of rapamycin (mTOR), a serine/threonine kinase, phosphorylates and modulates activity of S6K1 kinase [38-40]. The insulin activation of mTOR and S6K1 kinase works through the IRS-1/PI 3-kinase/Akt pathway, while amino acids seem to exert a direct effect on mTOR [41,42]. Activation of mTOR and S6K1 kinase leads to serine phosphorylation of IRS-1, with a subsequent decline in tyrosine phosphorylation of IRS-1 and IRS-1-associated PI 3-kinase activity, as discussed above. Although many mechanisms leading to serine phosphorylation of IRS proteins have been explored, the nutritional effect on this process in humans is not completely understood.

Second, a disruption in the balance between the amounts of the PI 3-kinase subunits may play a role in the development of insulin resistance [43,44]. This enzyme consists of a regulatory subunit, p85, and a catalytic subunit, p110 [45]. Normally, p85 monomer exists in excess to p110. Since the p85 subunit of PI 3-kinase directly binds to IRS-1, there exists a competition between the free p85 monomer and the p85-p110 heterodimer for the binding site on IRS-1. Since only the heterodimer is responsible for PI 3-kinase activity, increases or decreases in p85 expression could lead to increased or decreased PI 3-kinase activity in an inverse manner [46-49]. We have previously demonstrated that in the women who displayed reduced whole-body insulin sensitivity following 3 days of 50% overfeeding there was an increase in total p85α (the most abundant isoform of p85) expression in skeletal muscle. This increase in p85α expression was inversely correlated with PI 3-kinase activity [16].

We thus hypothesized that acute bouts of overnutrition would alter skeletal muscle insulin signaling prior to seeing changes in whole body insulin sensitivity. Moreover, we hypothesized that high fat overnutrition would result in greater impairment in insulin signaling than high carbohydrate overnutrition. The present study was designed to examine these hypotheses.

## Methods

### Subjects

Twenty-one lean (BMI 21.8 ± 1.8), healthy men (n = 11) and women (n = 10) with normal glucose tolerance (as determined by oral glucose tolerance testing) completed the study (Table 1). The study was approved by the Colorado Multiple Institutional Review Board, and all subjects gave informed consent.

**Table 1:** Baseline characteristics

**Characteristic**	**Value**
N (M/W)	21 (11/10)
Age (years)	27.8 ± 0.9
Weight (kg)	66.6 ± 1.5
BMI (kg/m^2^)	21.8 ± 0.4
Waist Circumference (cm)	78.7 ± 1.7
Fat Free Mass (kg)	51.0 ± 2.1
Percent Body Fat (%)	21.9 ± 1.9

Mean ± SD

### Materials

Bovine serum albumin (BSA) and protease inhibitors aprotinin and leupeptin were purchased from Boehringer Mannheim (Indianapolis, IN, USA). Antibodies to IRS-1, and p85α were purchased from Upstate Biotechnology (Lake Placid, NY, USA). Antibodies to p110 were purchased from Santa Cruz Biotechnology (Santa Cruz, CA, USA). Anti-phosphoserine IRS-1 antibody was from Upstate Biotechnology, pY-20 antibody was from BD Transduction Laboratories (San Diego, CA, USA). Antibodies to mTOR, phospho-mTOR, p70S6 kinase (S6K1) and phospho-p70S6 kinase (Thr 3389 and Thr421/Ser424) were from Cell Signaling Technology (Danvers, MA, USA). Secondary horseradish-peroxidase-conjugated antibody, protein A Sepharose and chemeluminescence detection reagent, ECLplus Western Blotting Analysis System, and ImageQuant TL software from GE Healthcare Bio Science/Amersham Biosciences, Piscataway, NJ. γ^32^P-ATP was purchased from Perkins-Elmer (Boston, MA). Whatman flexible plates for thin layer chromatography were obtained from Fisher Scientific (Denver, CO). Analytical grade resins, polyvinylidene difluoride membranes, PAGE gel equipment and protein assay kits were from Bio-Rad Laboratories (Hercules, CA, USA).

### Study Design

Subjects first underwent baseline assessments of resting metabolic rate (RMR) using the SensorMedics Vmax Spectra 29 metabolic cart (Sensormedics, Yorba Linda, CA) and body composition determined by dual x-ray absorptiometry (DEXA) using Hologic Discovery version 12.6. Subjects were then studied on three separate occasions with a one month wash-out between study visits. The first study day served as a baseline and was performed after subjects were fed a eucaloric diet (30% fat, 50% carbohydrate, 20% protein) for 5 days. Estimates of daily energy needs were made using several factors: 1) the Harris-Benedict equation, 2) baseline RMR plus an activity factor, and 3) lean body mass. Subjects were then studied after 5 days of high-carbohydrate (HC; 20% fat, 60% carbohydrate, 20% protein) and 5 days of high-fat (HF; 50% fat, 30% carbohydrate, 20% protein) overfeeding (40% caloric excess over a eucaloric diet) in a cross-over counter-balanced manner. Because the two hypercaloric diets contained 40% excess calories, carbohydrate intake on the HF diet and fat intake on the HC diet were similar to amounts consumed during eucaloric feeding (example: eucaloric 2000 kCal diet contained 66.7 g fat, 250 g CHO and 100 g protein; HC 2800 kCal diet contained 62.2 g fat, 420 g CHO and 140 g protein; HF 2800 kCal diet contained 155.6 g fat, 210 g CHO and 140 g protein). Dietary lipid for all diets contained a 1:1:1 ratio of monounsaturated, polyunsaturated and saturated fats. Food for all study diets (eucaloric, HC and HF) was provided by the Clinical and Translational Research Center (CTRC) kitchen. Subjects selected food choices from the CTRC kitchen menu (Additional File 1). The HC diet was enriched in fruits and starches. The HF diet was enriched in dairy, nuts, and oils. Subjects presented to the CTRC every morning to pick up food and be weighed. Subjects were asked to maintain their usual level of activity throughout the study. They were also asked not to consume any alcoholic or calorie-containing beverages.

### Study Days

Subjects were admitted to the inpatient CTRC the night of day 5 for each of the three diet phases for an overnight fast. The next morning a euglycemic-hyperinsulinemic clamp and skeletal muscle biopsy were performed. An antecubital venous catheter was placed in one arm for infusions, and another catheter was placed retrograde in a dorsal hand vein of the contralateral arm for sampling, using the heated hand technique to obtain arterialized venous blood. Glucose production and disposal were measured using a euglycemic-hyperinsulinemic clamp. After baseline blood samples were taken a primed (19 μmol/kg), constant (0.22 μmol/kg/min) infusion of [6,6-^2^H_2_] glucose was started to measure glucose disposal rate. A primed, continuous infusion of insulin at 40 mU/m^2^/min was then initiated and continued from time 120 to 240 minutes. Blood samples were taken at 90, 100, 110, 220, 230, and 240 min for steady state measurements of metabolites and isotope enrichments. Blood samples were taken every 5 minutes during the insulin infusion for bedside glucose analysis, and a 20% dextrose solution enriched with [6,6-^2^H_2_] glucose was infused and adjusted to maintain euglycemia at a blood glucose level of approximately 90 mg/dl. Rates of glucose appearance (Ra) and disappearance (Rd) were calculated using the modified Steele equation [50,51]. The skeletal muscle biopsy was performed once the insulin prime was complete (after 15 minutes) in order to assess insulin-stimulated effects on PI 3-kinase activity. Subcutaneous tissue overlying the vastus lateralis muscle was infiltrated with 1% lidocaine. A small incision was made with a scalpel down through the level of the fascia. A Bergstrom sidecut biopsy needle with suction was used to remove approximately 0.25 g of skeletal muscle tissue. Tissue samples were frozen immediately using the "freeze clamp" method. The biopsy samples were stored at -80°C until used.

### Determination of intramyocellular lipid content (IMCL)

In a subset of subjects (N = 10), intramuscular triglyceride content of the soleus muscle was measured via ^1^H (proton) magnetic resonance spectroscopy (MRS) performed using the 3.0 T whole-body MRI scanner (GE Medical Systems, Waukesha, WI). The spectroscopic acquisition is performed using the probe-p (PRESS) pulse sequence with parameters (TR/TE = 2000/100 ms, 128 averages, total acquisition time 7 minutes) optimized to avoid signals from fat. The resulting spectra are analyzed using LCModel software which fits the spectra using basis sets consisting of solution metabolite spectra.

### Clinical Laboratory Measurements

Plasma glucose level was measured throughout the euglycemic-hyperinsulinemic clamp studies at the bedside every 5 minutes using a YSI glucose analyzer (YSI Inc, Yellow Springs, OH). Baseline and steady-state analysis included blood sampling for insulin, glucose, free fatty acids (FFAs), glycerol, triglycerides, leptin, adiponectin, TNF-α and interleukin (IL)-6. Insulin was determined by radioimmunoassay (Diagnostic Systems Laboratory, Webster, TX). Serum glucose levels were determined using hexokinase, UV and triglycerides were determined by enzymatic assay (Olympus America, Inc., Center Valley, PA). FFAs were determined using an enzymatic assay (WaKo Chemicals, Richmond, VA). Glycerol was determined using an enzymatic assay (R-Biopharmm Inc., Marshall, MI). Leptin and adiponectin levels were determined by radioimmunoassays (Linco Research, Inc., St. Charles, MO). TNF-α and IL-6 were determined by ELISA (R&D Systems, Minneapolis, MN). Glucose isotopic enrichment was measured by gas chromatography/mass spectrometry (Metabolic Solutions, Inc., Nashua, NH).

### Western Blot Analysis

Immunoprecipitated IRS-1 was immunoblotted with pY20 and anti-serine 307 IRS-1 antibodies to determine extent of tyrosine and serine phosphorylation of IRS-1 as well as with anti-IRS-1 antibody for assessment of total IRS-1. Immunoprecipitated IRS-1 was also immunoblotted with p110 antibodies to determine the total amount of IRS-1-associated p110 expression. Tissue homogenates were immunoprecipitated and immunoblotted with p85α specific antibody for assessment of p85α protein expression. Finally, homogenates were also immunoprecipitated with mTOR and S6K1 specific antibodies and then blotted with phospho-mTOR (serine 2448) and phospho-S6K1 kinase (serine 371) antibodies, respectively. The total amounts of mTOR and S6k1 kinase were determined by immunoblotting with corresponding specific antibodies.

### Determination of IRS-1-associated PI 3-kinase activity

Lysates prepared from the tissue biopsy were immunoprecipitated with IRS-1 antibody. PI 3-kinase activity is determined in 1 to 3 μL of the immunoprecipitate by the thin layer chromatography as described in several of our publications [52].

### Statistical Analysis

Data are presented as mean ± SEM. Statistical analysis were done using SigmaStat software (Jandel Scientific, San Rafael, CA, USA). The effects of study diets were analyzed using repeated measures analysis of variance (ANOVA). *p*-values of < 0.05 were considered statistically significant. No gender differences were found, so all results reported include data from men and women combined as one cohort.

## Results

### In Vivo

After 5 days of eucaloric (EC) feeding, baseline whole-body insulin sensitivity and hepatic glucose production were determined by euglycemic-hyperinsulinemic clamp. Subjects were then studied after 5 days of high-carbohydrate (HC) and high-fat (HF) overfeeding in a counter balanced manner. There was no difference in weight between study days (EC: 66.6 ± 1.6 kg; HC: 67.5 ± 1.5 kg, HF: 67.2 ± 1.6 kg, p = 0.92). HC overfeeding resulted in increased fasting insulin and triglyceride concentrations and lower fasting free fatty acid concentrations as compared to EC feeding (Table 2). HF overfeeding was associated with a significant decrease in triglyceride concentrations compared to EC and HC feedings (Table 2).

**Table 2:** Fasting metabolic data

	**EC**	**HC**	**HF**
Insulin (mU/mL)	4.2 ± 0.4	5.8 ± 0.6*	4.4 ± 0.4
Glucose (mg/dL)	84.2 ± 1.3	85.5 ± 1.7	84.9 ± 1.4
FFA (μEq/L)	447 ± 41	315 ± 32.5*	404 ± 25.3
Triglycerides (mg/dL)	69.1 ± 5.9	91.0 ± 11.0**	60.6 ± 7.9^#^
Glycerol (μM/L)	72.1 ± 7.8	73.2 ± 11.4	69.6 ± 8.9
Adiponectin (μg/mL)	10.0 ± 1.2	9.9 ± 1.1	9.5 ± 1.2
Leptin (ng/mL)	6.2 ± 1.6	8.3 ± 2.1	6.5 ± 1.5
TNF-α (pg/mL)	2.1 ± 0.4	2.2 ± 0.5	2.0 ± 0.4
Interleukin-6 (pg/mL)	0.9 ± 0.1	0.9 ± 0.1	1.3 ± 0.5

Mean ± SE.

EC = eucaloric diet, HC = high-carbohydrate overfeeding, HF = high-fat overfeeding

*p < 0.03 compared to EC, **p < 0.001 compared to EC, #*p *< 0.001 compared to HC

Interestingly, five days of HC or HF overfeeding did not alter whole-body insulin sensitivity compared to eucaloric feeding, expressed as either M-Value or GDR (Table 3). Steady-state serum glucose and insulin levels were equivalent at the end of the euglycemic-hyperinsulinemic clamp for all three study days (Table 3). There was also no significant change in basal hepatic glucose output following either overfeeding diet (Table 3).

**Table 3:** Euglycemic-hyperinsulinemic clamp data

	**EC**	**HC**	**HF**
M-Value (mg/kg FFM-min)	11.4 ± 2.7	11.2 ± 2.9	11.1 ± 2.1
GDR (mg/kg FFM-min)	12.1 ± 4.7	10.9 ± 2.7	10.8 ± 3.4
Steady State Glucose (mg/dL)	79.2 ± 5.1	81.8 ± 8.7	79.6 ± 8.8
Steady State Insulin (mU/mL)	66.8 ± 22.3	60.2 ± 17.4	59 ± 27.1
MCR (mL/m^2^-min)	719 ± 263.5	785 ± 175.2	825 ± 232.6
Rest HGO (mg/kg FFM-min)	3.39 ± 0.5	3.79 ± 1.5	3.42 ± 1.0

Mean ± SE.

EC = eucaloric diet, HC = high-carbohydrate overfeeding, HF = high-fat overfeeding

GDR = glucose disposal rate, MCR = metabolic clearance rate of insulin, HGO = hepatic glucose output

Measurements of serum adipokines and markers of systemic inflammation revealed no significant change in serum adiponectin or leptin levels following overfeeding with either HC or HF diets. Similarly, there was no significant change in TNFα or interleukin-6 levels following either overfeeding diet (Table 2).

Finally, assessment of intramyocellular triglyceride content (IMCL) performed in a subset of subjects (N = 10) following both HC and HF overfeeding demonstrated 17-22% increases in IMCL accumulation after both diets (EC: 1077.0 ± 103.2, HC: 1323.3 ± 137.6 *p *= 0.0025, HF: 1268.8 ± 128.9 *p *= 0.0136).

### Ex Vivo

**Figure 1:**
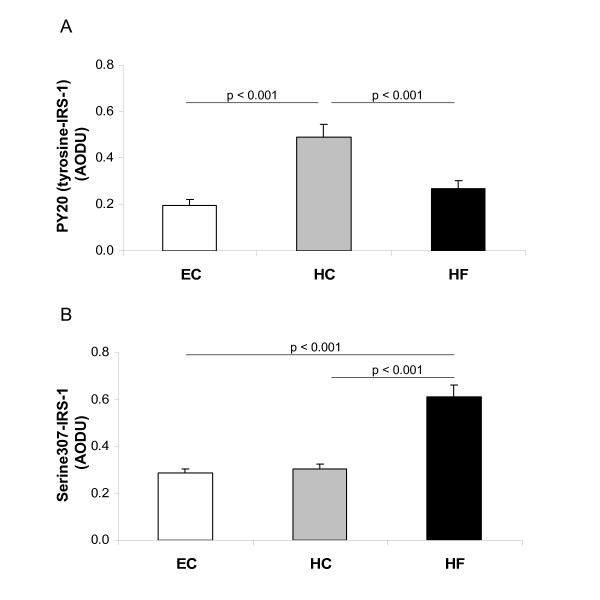
**Phosphorylation of IRS-1**. Tyrosine (A) and Serine (B) phosphorylation of IRS-1 in skeletal muscle following Eucaloric feeding (EC), high-carbohydrate overfeeding (HC), and high-fat overfeeding (HF). HC overfeeding increased tyrosine phosphorylation of IRS-1 compared to EC feeding. HF overfeeding increased serine phosphorylation of IRS-1 compared to EC feeding.

**Figure 2:**
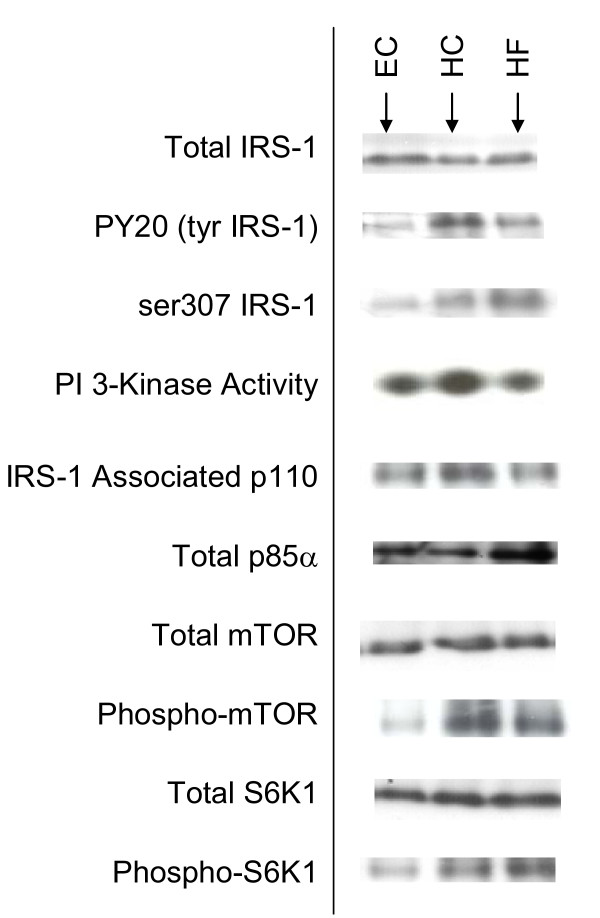
**Representative blots**. Representative blots from *ex vivo *skeletal muscle tissue following Eucaloric feeding (EC), high-carbohydrate overfeeding (HC), and high-fat overfeeding (HF).

We then examined ex vivo markers of insulin signaling in skeletal muscle of individuals enrolled into this study. All biopsies were obtained at the end of each feeding period in the insulin-stimulated condition. Five days of HC overfeeding resulted in significant increases in tyrosine phosphorylation of IRS-1 (Figure 1A), with no change in total IRS-1 protein expression compared to EC feeding (Figure 2). In contrast, HF overfeeding resulted in increased serine phosphorylation of IRS-1 (Figure 1B) with no change in the amount of total IRS-1 (Figure 2). Representative blots of total and phosphorylated IRS-1 are shown in Figure 2 along with all subsequent blots discussed below.

**Figure 3:**
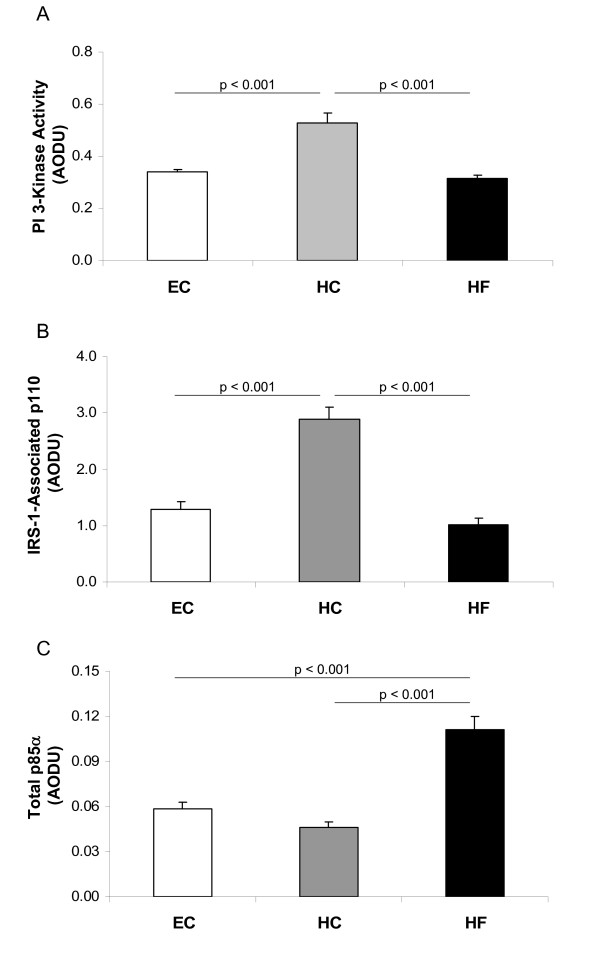
**PI 3-kinase activity and subunit expression**. IRS-1-associated PI 3-kinase activity (A), IRS-1-associated p110 protein expression (B), and Total p85α protein expression (C) in skeletal muscle following Eucaloric feeding (EC), high-carbohydrate overfeeding (HC), and high-fat overfeeding (HF). HC overfeeding increased IRS-1 associated PI 3-kinase activity and IRS-1 associated p110 expression compared to EC feeding. HF overfeeding increased total p85α expression compared to EC feeding.

In concert with these changes in IRS-1 phosphorylation, insulin-stimulated IRS-1-associated PI 3-kinase activity increased following HC overfeeding (Figure 3A). PI 3-kinase is a heterodimer comprised of regulatory, p85, and catalytic, p110, subunits. While p85 is responsible for association of PI 3-kinase with IRS-1, it is actually association of p110 with IRS-1 that determines PI 3-kinase activity. Therefore, we examined association of p110 with IRS-1 in response to insulin in patients after 5 days of overfeeding either HC or HF compared to 5 days of eucaloric feeding. The IRS-1 associated p110 was significantly increased following HC overfeeding (Figure 3B), supporting a positive influence of the HC diet on PI 3-kinase activity.

Decreased association of PI 3-kinase with IRS-1 can be a consequence of either serine phosphorylation of IRS-1 or of an increased expression of p85 monomer that competes with p85-p110 heterodimer for the IRS-1 binding sites. Therefore, we examined total amounts of p85α (a predominant isomer of p85) after overfeeding HC and HF. Total p85α protein expression was increased following HF overfeeding compared to eucaloric feeding leading to enhanced competition with PI 3-kinase heterodimer, thus contributing to the reduction in IRS-1 associated p110 levels (Figure 3C). In contrast, p85α levels were lower following HC overfeeding compared to HF overfeeding, thus maintaining minimal competition with PI 3-kinase for the IRS-1 binding sites (Figure 3C).

**Figure 4:**
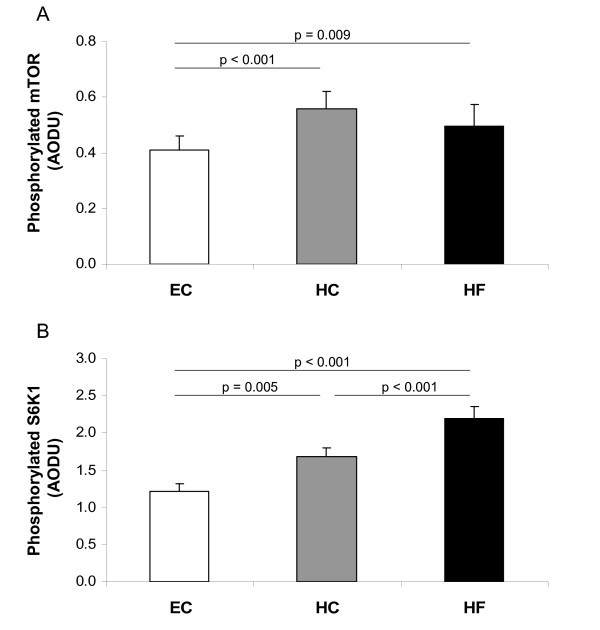
**mTOR and S6K1 activity**. Phosphorylation of mTOR (A) and S6K1 (B) in skeletal muscle following Eucaloric feeding (EC), high-carbohydrate overfeeding (HC), and high-fat overfeeding (HF). Both HC and HF overfeeding increased phosphorylation of mTOR and S6K1 compared to EC feeding.

Looking further downstream from PI 3-kinase, we assessed changes in the nutrient sensor, mTOR, and its effect on S6K1 kinase. Total protein expression of mTOR and S6K1 kinase was unchanged following the study diets (Figure 2). However, there was a significant increase in phosphorylated mTOR resulting in increased phosphorylation of S6K1 following both overfeeding diets (Figure 4).

## Discussion

The salient feature of the current study is that short-term overfeeding in healthy lean individuals (40% caloric excess for 5 days) results in significant changes in skeletal muscle insulin signaling before any alterations in total body insulin sensitivity are evident. Furthermore, macronutrient composition of the overfeeding diet has a profound influence on changes in insulin signaling in skeletal muscle.

We found that consuming a high-carbohydrate (HC) hypercaloric diet results in a significant increase in tyrosine phosphorylation IRS-1, with increased association of p110 (a catalytic subunit of PI 3-kinase) with IRS-1 and enhanced IRS-1-associated insulin-stimulated PI 3-kinase activity. These changes in insulin signaling usually denote increased insulin sensitivity and may be a result of the modest hyperinsulinemia seen with HC overfeeding. Whole body insulin sensitivity as measured by the euglycemic-hyperinsulinemic clamp, however, did not change after 5 days of overfeeding, suggesting the presence of early changes in insulin signaling in response to a high-carbohydrate load directed at better disposal of this load in order to maintain whole-body insulin sensitivity.

In contrast, we found that high-fat (HF) overfeeding results in a significant increase in serine phosphorylated IRS-1, a traditional determinant of insulin resistance. At the same time, HF overfeeding is associated with increased expression of p85α and decreased association of p110 with IRS-1 and decreased insulin-stimulated PI 3-kinase activity. Although in the present study we cannot determine which component (serine phosphorylation of IRS-1 or increased expression of p85α) plays a greater role, these changes are typically associated with insulin resistance in skeletal muscle. In our previous studies, overfeeding healthy female subjects with 50% caloric excess for 3 days, we also observed an increase in expression of p85α before serine phosphorylation of IRS-1 [16], suggesting that excess energy intake may drive overexpression of p85α as an earliest molecular change in response to overfeeding. As with HC overfeeding, these ex-vivo alterations were not accompanied by any change in the in vivo assessment of insulin sensitivity. Excess fat intake appears to alter carbohydrate induced insulin signaling at the level of skeletal muscle but without an appreciable change in whole body insulin sensitivity. These findings again imply the appearance of early changes to acute bouts of overnutrition; however, the effects vary depending on the macronutrient composition of the diet.

Assessing effects further downstream of PI 3-kinase, we found that both HC and HF overfeeding led to significant increases in activation of the nutrient sensor, mTOR, and its downstream target, S6K1. Ability of S6K1 to promote serine phosphorylation of IRS-1 has been suggested as a potential mechanism of insulin resistance [30-37,53,54]. In this study, both overfeeding diets induced significant increases in phosphorylation of mTOR and S6K1, yet only HF overfeeding was associated with increased serine phosphorylation of IRS-1. Further studies are needed to evaluate this discrepancy.

Interestingly, in a subset of subjects, we found a significant increase in intramyocellular lipid content (IMCL) following both HC and HF overfeeding compared to baseline. This increase was observed regardless of the macronutrient content of the diet. This increase in IMCL was also seen in the setting of unchanged whole-body insulin sensitivity, suggesting that either IMCL is not associated with insulin sensitivity, the duration of the study was not sufficient to see an effect, or the type or source of myocellular lipid may be important. Although literature suggests that increases in intramuscular triglyceride are associated with increased insulin resistance [55-57], some data suggest these may not be related [58,59]. Additionally, studies have shown that the source of intramyocellular lipid may determine how the lipid accumulation affects insulin responsiveness. Ceremide and diacylglycerol (DAG) have been linked to deleterious effects on insulin signaling in muscle and DAG levels can increase following intake of a diet high in saturated fat [60,61]. In contrast, triacylglycerol fatty acids (TAG) accumulate in muscle following a diet high in poluyunsaturated fatty acids and can lead to improved insulin sensitivity [61]. Clearly further studies are needed to understand the relationship and interaction between IMCL and insulin signaling. Future studies would be strengthened by performing direct measurements of intramyocellular lipid metabolites.

There are a few limitations to this study that need to be discussed. First, the duration and amount of overfeeding were chosen to be 5 days and 40%, respectively. It is possible that a longer duration of overnutrition is necessary to impact whole-body insulin sensitivity. Brøns, et al. recently published data where they also did not see any appreciable difference in whole-body insulin sensitivity, as measured by euglycemic-hyperinsulinemic clamp following 5 days of 50% caloric excess feeding [17]. Conceivably, changes in the whole-body insulin sensitivity may not be seen either until an individual begins to show clinically significant weight gain or there is a more significant caloric excess. Studies examining longer term overfeeding should be performed to further assess this issue. Second, despite strict eligibility criteria there was significant heterogeneity in baseline measures of insulin sensitivity (M-value: 7.68 to 17.71 mg/kgFFM/min). While we did not see an association between baseline insulin sensitivity and responses to overfeeding there may be differences in how individuals can or cannot respond to overnutrition based on their baseline insulin sensitivity. Studies in larger cohorts of subjects might be needed to uncover changes in whole-body insulin sensitivity following overnutrition.

## Conclusion

We conclude that acute bouts of overnutrition lead to early changes at the cellular level before whole-body insulin sensitivity is altered. Our lean healthy cohort of subjects may be metabolically flexible and thus able to adapt to such changes in their diet. High carbohydrate overfeeding induced mild elevations in insulinemia and triglyceridemia, while still suppressing FFA, hepatic glucose production and stimulating glucose disposal. On a signaling level, HC overfeeding induced changes compatible with increased insulin sensitivity. In contrast, molecular changes in HF overfeeding were compatible with a reduced insulin sensitivity, while in vivo insulin sensitivity remained unchanged. More studies are needed to determine when these early responses can no longer sustain normal whole-body insulin sensitivity and which individuals may not be as capable of adapting to overnutrition and why.

## Competing interests

The authors declare that they have no competing interests.

## Authors' contributions

RLA conducted euglycemic-hyperinsulinemic clamps, performed skeletal muscle biopsies, analyzed *in vivo *data, performed the statistical analysis and drafted the manuscript. JWL performed all *ex vivo *analysis of skeletal muscle tissue. KG was the study coordinator, organized all subject visits and assisted with euglycemic-hyperinsulinemic clamps. BD co-conceived of the study, and participated in its design and coordination, assisted with data analysis and helped draft the manuscript. MAC co-conceived of the study, and participated in its design and coordination, conducted euglycemic-hyperinsulinemic clamps, performed skeletal muscle biopsies, assisted with data analysis and statistical analysis and helped draft the manuscript. All authors read and approved the final manuscript.

## Supplementary Material

Additional file 1 **Examples of Diets**. The data provided represent an example of the types and amounts of foods for each diet for one subject. Click here for file
