# Supplementary material for: Early responses of insulin signaling to high-carbohydrate and high-fat overfeeding
Source: Nutr Metab (Lond). 2009 Sep 28;6:37. doi: 10.1186/1743-7075-6-37 (PMC2761378; doi:10.1186/1743-7075-6-37)
Supplement: Additional file 1 — Examples of Diets. The data provided represent an example of the types and amounts of foods for each diet for one subject. [file 1743-7075-6-37-S1.DOC]

Examples of Diets

| Day 1 |  | | |  | | |  | | |
| --- | --- | --- | --- | --- | --- | --- | --- | --- | --- |
|  | **Eucaloric Diet** | | | **High Fat Overfeeding** | | | **High Carb Overfeeding** | | |
| Meal | Food Description | Amount | Food Description | | Amount | Food Description | | Amount |  |
| Breakfast | Banana | 90g | Cantaloupe | | 120g | Banana | | 95g |  |
|  | Orange Juice | 1 box | Oil Olive | | 15g | Orange Juice | | 2 boxes |  |
|  | Oil Olive | 10g | Cheese Cheddar Shredded | | 20g | Oil Olive | | 12g |  |
|  | Peanut Butter Smooth | 30g | Margarine Canola Harvest | | 10g | Cheese Cheddar FF Kraft | | 30g |  |
|  | Eggbeaters | 120g | Egg Liquid Whole | | 180g | Eggbeaters | | 200g |  |
|  | Margarine Canola Harvest | 10g | Bagel Plain | | 80g | Margarine Canola Harvest | | 10g |  |
|  | Bagel Plain | 80g |  | |  | Ham , smoked (water added, honey) | | 90g |  |
|  |  |  |  | |  | Bagel Plain | | 80g |  |
| Lunch | Grapes Red or Green | 120g | Cheese Swiss | | 30g | Turkey Breast | | 160g |  |
|  | Lettuce Looseleaf | 2 each | Mayonnaise Kraft | | 1 each | Lettuce Looseleaf | | 2 each |  |
|  | Tomato Raw | 2 slices | Peanut Butter Smooth | | 30g | Tomato Raw | | 2 slices |  |
|  | Cheese American FF Kraft | 2 slices | Lettuce Looseleaf | | 1 each | Cheese American FF Kraft | | 2 slices |  |
|  | Mayonnaise Light Kraft | 2 each | Tomato Raw | | 1 slice | Mayonnaise Light Kraft | | 2 each |  |
|  | Bread Wheat Home Pride | 110g | Bread Wheat Home Pride | | 58g | Potato Chip BAKED Plain | | 1 package |  |
|  | Ham , smoked (water added, honey) | 140g | Ritz Cracker | | 6 each | Fig Newton-single stack cookie | | 4 each |  |
|  | Doritos | 1 package | Ham , smoked (water added, honey) | | 150g | Bread Wheat Home Pride | | 110g |  |
|  | Mustard, Spicy Brown | 2 each | Doritos | | 1 package | Mustard, Spicy Brown | | 2 each |  |
|  |  |  | Mustard, Spicy Brown | | 1 each | Grape Juice | | 2 cans |  |
|  |  |  | Peanuts Planters | | 1 package |  | |  |  |
| Supper | Parmesan Cheese | 5g | Parmesan Cheese | | 5g | Parmesan Cheese | | 8g |  |
|  | Chicken Breast | 110g | Beef Ground Extra Lean | | 145g | Chicken Breast | | 140g |  |
|  | Oil Olive | 8g | Strawberries Fresh | | 100g | Apple Juice | | 2 boxes |  |
|  | Broccoli | 120g | Oil Olive | | 10g | Oil Olive | | 12g |  |
|  | Sherbet | 1 each | Oil Olive | | 16g | Broccoli | | 90g |  |
|  | Margarine Canola Harvest | 1g | Broccoli | | 120g | Sherbet | | 2 each |  |
|  | Spaghetti COOKED | 190g | Ice Cream Vanilla | | 1 each | Cheese Cheddar FF Kraft | | 28g |  |
|  | Classico Pasta Sauce | 170g | Cheese Cheddar FF Kraft | | 32g | Margarine Canola Harvest | | 8g |  |
|  | Roll, Dinner, Sara Lee | 1 each | Margarine Canola Harvest | | 5g | Spaghetti COOKED | | 250g |  |
|  |  |  | Spaghetti COOKED | | 200g | Classico Pasta Sauce | | 250g |  |
|  |  |  | Classico Pasta Sauce | | 150g | Roll, Dinner, Sara Lee | | 2 each |  |
|  |  |  | Roll, Dinner, Sara Lee | | 1 each |  | |  |  |
| Snack | Yogurt Light Blueberry Dannon | 1 each | Tuna Light Starkist Drained | | 1 can | Grapes Red or Green | | 185g |  |
|  | Peanut Butter Cheese Crackers 4/PK | 1 each | Mayonnaise Kraft | | 1 each | Starburst Chews | | 8 each |  |
|  |  |  | Celery | | 1 each | Yogurt Light Rasp Dannon | | 1 each |  |
|  |  |  | Triscuits Nabisco | | 10 each |  | |  |  |

| Day 2 |  | | |  | | |  | |
| --- | --- | --- | --- | --- | --- | --- | --- | --- |
|  | **Eucaloric Diet** | | | **High Fat Overfeeding** | | | **High Carb Overfeeding** | |
| Meal | Food Description | Amount | Food Description | | Amount | Food Description | | Amount |
| Breakfast | Strawberries Fresh | 110g | Oil Olive | | 15g | Cranberry Juice Cocktail | | 2 boxes |
|  | Orange Juice | 2 boxes | Cheese Cheddar Shredded | | 30g | Cheese Cheddar FF Kraft | | 30g |
|  | Oil Olive | 11g | Honeydew | | 110g | Pears in Juice | | 2 each |
|  | Cheese Cheddar FF Kraft | 20g | Margarine Canola Harvest | | 8g | Waffle Eggo Homestyle | | 2 each |
|  | Syrup Cary SUGAR FREE | 30g | Egg Liquid Whole | | 180g | Eggbeaters | | 200g |
|  | Waffle Eggo Homestyle | 2 each | Yogurt Light Rasp Dannon | | 1 each | Margarine Canola Harvest | | 6g |
|  | Eggbeaters | 170g | English Muffin | | 56g | Syrup Pancake | | 40g |
|  | Margarine Canola Harvest | 6g | Ham , smoked (water added, honey) | | 80g |  | |  |
| Lunch | Lettuce Looseleaf | 1 each | Turkey Breast | | 115g | Pineapple Tidbits (juice pack) | | 2 each |
|  | Tomato Raw | 2 slices | Peanut Butter Smooth | | 30g | Lettuce Looseleaf | | 2 each |
|  | Cheese American FF Kraft | 2 slices | Lettuce Looseleaf | | 1 each | Tomato Raw | | 2 slices |
|  | Mayonnaise Light Kraft | 1 each | Tomato Raw | | 1 slice | Cheese American FF Kraft | | 2 slices |
|  | Pretzels Rold Gold | 1 package | Carrot Baby | | 70g | Mayonnaise Light Kraft | | 2 each |
|  | Roast Beef Deli Slices | 90g | Mayonnaise Light Kraft | | 1 each | Pretzels Rold Gold | | 1 package |
|  | Bread Wheat Home Pride | 56g | Dressing Ranch KRAFT | | 20g | Gatorade | | 1 each |
|  | Mustard, Spicy Brown | 1 each | Sunchips Cheddar | | 1 package | Yogurt Light Blueberry Dannon | | 1 each |
|  | Peanuts Planters | 30g | Saltine Cracker | | 3 packages | Bread Wheat Home Pride | | 110g |
|  |  |  | Cheese Cheddar Sliced | | 30g | Ham , smoked (water added, honey) | | 150g |
|  |  |  | Bread Wheat Home Pride | | 58g | Mustard, Spicy Brown | | 2 each |
|  |  |  | Mustard, Spicy Brown | | 1 each | Cereal Bar Nutrigrain | | 1 each |
| Supper | Milk Skim | 1 box | Chicken Breast | | 140g | Milk Skim | | 1 box |
|  | Chips Ahoy | 4 each | Oil Olive | | 24g | Applesauce Natural | | 2 each |
|  | Peppers Green or Red | 4 slices | Potato Baked Flesh | | 200g | Oil Olive | | 13g |
|  | Dressing Ranch KRAFT | 20g | Chips Ahoy | | 5 each | Potato Baked Flesh | | 200g |
|  | Cheese Cheddar FF Kraft | 20g | Cheese Cheddar Shredded | | 30g | Corn | | 120g |
|  | Margarine Canola Harvest | 6g | Peppers Green or Red | | 3 slices | Chips Ahoy | | 4 each |
|  | Lettuce Romaine | 2 cups | Margarine Canola Harvest | | 6g | Margarine Canola Harvest | | 6g |
|  | LC Pizza Pepperoni | 1 box | Lettuce Romaine | | 2 cups | Pork Chop | | 180g |
|  | Roll, Dinner, Sara Lee | 1 each | Vinegar Balsamic | | 15g | Roll, Dinner, Sara Lee | | 2 each |
|  |  |  | Walnuts, English | | 30g |  | |  |
|  |  |  | Roll, Dinner, Sara Lee | | 1 each |  | |  |
|  |  |  | Lettuce Looseleaf | | 1 each |  | |  |
| Snack | Tuna Light Starkist Drained | 1 can | Tomato Raw | | 1 slice | Tuna Light Starkist Drained | | 1 can |
|  | Celery | 1 each | Mayonnaise Light Kraft | | 1 each | Apple Juice | | 2 boxes |
|  | Mayonnaise FF Kraft | 1 each | Roast Beef Deli Slices | | 100g | Celery | | 1 each |
|  | Triscuits Nabisco | 8 each | Cheese Cheddar Sliced | | 30g | Mayonnaise FF Kraft | | 1 each |
|  | Gatorade | 1 each | Bread Wheat Home Pride | | 56g | Triscuits Nabisco | | 10 each |
|  |  |  | Potato chip Lays | | 1 package |  | |  |
